# Physalin H, physalin B, and isophysalin B suppress the quorum-sensing function of *Staphylococcus aureus* by binding to AgrA

**DOI:** 10.3389/fphar.2024.1365815

**Published:** 2024-04-10

**Authors:** Junpei Yamaguchi, Teruhisa Manome, Yasumasa Hara, Yuriko Yamazaki, Yuumi Nakamura, Masami Ishibashi, Akiko Takaya

**Affiliations:** ^1^ Department of Infection Control Science, Graduate School of Pharmaceutical Sciences, Chiba University, Chiba, Japan; ^2^ Graduate School of Pharmaceutical Sciences, Chiba University, Chiba, Japan; ^3^ Laboratory of Natural Products Chemistry, Faculty of Pharmaceutical Sciences, Hokkaido University, Sapporo, Japan; ^4^ Faculty of Agriculture, Kagawa University, Takamatsu, Japan; ^5^ Cutaneous Allergy and Host Defense, Immunology Frontier Research Center, Osaka University, Osaka, Japan; ^6^ Department of Dermatology, Graduate School of Medicine, Osaka University, Osaka, Japan; ^7^ School of Pharmacy at Fukuoka, International University of Health and Welfare, Okawa, Japan; ^8^ Medical Mycology Research Center, Chiba University, Chiba, Japan; ^9^ Plant Molecular Science Center, Chiba University, Chiba, Japan

**Keywords:** physalins, MRSA, Agr-QS modulator, anti-hemolytic activity, AgrA-DNA inhibition, molecular docking, molecular dynamics simulation

## Abstract

The virulence of *Staphylococcus aureus*, including methicillin-resistant *S. aureus* (MRSA), depends on the expression of toxins and virulence factors controlled by the quorum-sensing (QS) system, encoded on the virulence accessory gene regulator (*agr*) locus. The aim of this study was to identify a phytochemical that inhibits Agr-QS function and to elucidate its mechanism. We screened 577 compounds and identified physalin H, physalin B, and isophysalin B—–phytochemicals belonging to physalins found in plants of the Solanaceae family—–as novel Agr-QS modulators. Biological analyses and *in vitro* protein–DNA binding assays suggested that these physalins suppress gene expression related to the Agr-QS system by inhibiting binding of the key response regulator AgrA to the *agr* promoters, reducing the function of hemolytic toxins downstream of these genes in MRSA. Furthermore, although physalin F suppressed gene expression in the Agr-QS system, its anti-hemolytic activity was lower than that of physalins H, B, and isophysalin B. Conversely, five physalins isolated from the same plant with the ability to suppress Agr-QS did not reduce bacterial Agr-QS activity but inhibited AgrA binding to DNA *in vitro*. A docking simulation revealed that physalin interacts with the DNA-binding site of AgrA in three docking states. The carbonyl oxygens at C-1 and C-18 of physalins, which can suppress Agr-QS, were directed to residues N201 and R198 of AgrA, respectively, whereas these carbonyl oxygens of physalins, without Agr-QS suppression activity, were oriented in different directions. Next, 100-ns molecular dynamics simulations revealed that the hydrogen bond formed between the carbonyl oxygen at C-15 of physalins and L186 of AgrA functions as an anchor, sustaining the interaction between the carbonyl oxygen at C-1 of physalins and N201 of AgrA. Thus, these results suggest that physalin H, physalin B, and isophysalin B inhibit the interaction of AgrA with the *agr* promoters by binding to the DNA-binding site of AgrA, suppressing the Agr-QS function of *S. aureus*. Physalins that suppress the Agr-QS function are proposed as potential lead compounds in the anti-virulence strategy for MRSA infections.

## 1 Introduction

*Staphylococcus aureus*, a Gram-positive bacterium, is the causal agent underlying several community and hospital-acquired infections. In 2019, *S. aureus* was responsible for more than 700,000 deaths globally, including 100,000 deaths associated with antibacterial-resistant strains of *S. aureus* (Antimicrobia Resistance, 2022). Methicillin-resistant *S. aureus* (MRSA) has multidrug resistance, is highly pathogenic, and frequently causes infection outbreaks; therefore, the increased prevalence of drug-resistant bacteria has become a global health problem that needs to be urgently addressed (Turner et al., 2019).

To establish infection, *S. aureus* deploys an array of virulence factors, depending on its growth phase (Novick, 2003; Wang and Muir, 2016). The virulence of *S. aureus* relies, in part, on the quorum sensing (QS) system, a mechanism of gene regulation in which bacteria coordinate the expression of certain genes in accordance with the concentration of small signal molecules (Defoirdt et al., 2013). A master regulator is encoded by the virulence accessory gene regulator (*agr*) locus on its genome (Patel and Rawat, 2023; Williams et al., 2023). The *agr* locus comprises divergent transcriptional units controlled by the P2 and P3 promoters. The P2 operon encodes *agrBDCA*, whose expression responds to an autoinducer peptide (AIP) (Novick et al., 1995; Novick and Geisinger, 2008). The P3 operon controls the expression of the regulatory RNA effector, RNAIII, which drives the transition from a sticky to a poisonous phenotype (Novick et al., 1993). A pro-peptide translated from the *agrD* transcript synthesizes AIP via AgrB-dependent proteolytic processing (Kavanaugh et al., 2007). AIP is transported through the cell membrane by AgrB and acts as an extracellular signaling molecule for QS (Zhang and Ji, 2004). AgrC and AgrA constitute the two-component signal transduction system, with AgrC being auto-phosphorylated upon the binding of AIP (Lina et al., 1998). The extracellular concentration of AIP increases with bacterial density, and AIP subsequently induces trans-phosphorylation of the response regulator AgrA by AgrC (Novick et al., 1995). Phosphorylated AgrA can bind to the P2 and P3 promoters and regulate the expression of downstream genes, including virulence factors.

Agr-QS regulates toxins and other factors involved in *S. aureus* pathogenesis (Cheung et al., 2011; Cheung et al., 2021); therefore, compounds that inhibit Agr-QS have been investigated as an alternative therapeutic strategy to antibiotics (Otto, 2023). *S. aureus* possesses four subgroups of Agr-QS—I, II, III and IV—which differ in the amino acid sequence of AIP; different AIPs cross-inhibit AIP–AgrC interactions. AIP produced by staphylococci such as *Staphylococcus epidermidis* also inhibits *S. aureus* Agr-QS (Ji et al., 1997). Therefore, compounds containing structural analogs of AIP inhibit Agr-QS by inhibiting the interaction between AgrC and AIP (Mayville et al., 1999; Mansson et al., 2011; Piewngam et al., 2018). Regarding other targets of Agr-QS inhibition, small compounds that interact with AgrA have been identified. For example, savirin was identified from 24,087 compounds using whole-cell P3 promoter screens. It was found to disrupt Agr-QS by inhibiting AgrA–DNA binding in MRSA, but not in skin commensal *S. epidermidis* (Sully et al., 2014). Small compounds such as diflunisal, hispidulin, and azan-7, which were identified through biological screening, also inhibit the binding of AgrA and DNA (Khodaverdian et al., 2013; Bernabe et al., 2021; Ren et al., 2022). Recently, compounds that inhibit the binding of AgrA to DNA have been proposed based on computer analyses predicting their structural interaction with AgrA (Ramasamy et al., 2023).

Plant species produce a wide range of secondary metabolites that provide a chemical line of defense against environmental microorganisms. Many phytochemicals have been identified that modulate the production of bacterial virulence factors and have potential to become lead compounds in research on anti-virulence therapy (Silva et al., 2016). Physalins, or 16,24-cyclo-13,14-seco steroids, belong to the class of withanolides that exhibit promising pharmacological properties (Wu et al., 2021; Yang et al., 2022). More than 78 different chemical structures of physalins, which are mainly produced by the genus *Physalis* spp. (Solanaceae), have been described over the past 50 years (Wu et al., 2021). Although no physalin has been fully synthesized to date, various biological functions such as antitumor activity (Antoun et al., 1981; Damu et al., 2007), anti-inflammatory activity (Vieira et al., 2005), and inhibition of NF-*κ*B signaling (Jacobo-Herrera et al., 2006) have been revealed using extracted physalins. Furthermore, studies have investigated the antibacterial activity of physalins. For example, physalin B was found to possess antimicrobial activity against *S. aureus* (Silva et al., 2005), while physalin D inhibited the growth of *S. aureus*, *S. epidermidis*, *Enterococcus faecalis*, and *Bacillus subtilis* with a minimum inhibitory concentration (MIC) of 32–64 *μ*g/mL (Helvaci et al., 2010). A crude fraction containing physalins B, F, and D possessed antimycobacterial activity against the *Mycobacterium tuberculosis* H37Rv strain (Januario et al., 2002). However, it remains unclear whether physalins bind to specific bacterial proteins and alter bacterial cell activity.

Previously, we have isolated natural compounds mainly derived from Southeast Asian herbal medicines (Arai et al., 2021). In this study, to determine phytochemicals with the Agr-QS inhibition activity, we screened 577 compounds. The ability of the compounds to suppress Agr-QS was analyzed using a whole-cell P3 reporter strain in MRSA. We investigated the Agr-QS activity of physalin H obtained via screening and its ability to suppress the virulence of *S. aureus* using several biological assays. Furthermore, we attempted to reveal the structure of physalins required for Agr-QS inhibition by comparing the biological activity and *in vitro* AgrA–DNA inhibitory assay with eight physalins isolated from *Physalis minima* in addition to physalin H (Manome et al., 2023). Molecular docking and dynamics simulations were performed to understand how physalins that inhibit Agr-QS interact with AgrA.

## 2 Materials and methods

### 2.1 Bacterial strains and growth conditions

The following *S. aureus* strains were used in this study: USA 300 strain LAC (Agr-QS subgroup I) and P3-*luc* (*agr*:P3-*luc*) (Nakamura et al., 2013); M1K051 (Agr-QS subgroup I, MSSA) and M1K155 (Agr-QS subgroup II, MSSA) (Nakamura et al., 2020); CN02 (Agr-QS subgroup III, MRSA) was isolated at Chiba University Hospital, Japan. *S. aureus* was cultured in Tryptic soy broth (TSB) (Millipore, USA) overnight at 37°C with shaking before use in the experiments. Frozen *S. aureus* stocks were maintained at −80°C in TSB supplemented with 40% glycerol. When counting colony-forming unit (CFU), samples were serially diluted in phosphate-buffered saline and plated on TSB agar plates. CFU counting was performed 18–24 h later. *Escherichia coli* DH5*α*Z1 was grown in Luria-Bertani (LB) medium. When introducing a plasmid, the LB medium was supplemented with 100 *μ*g/mL ampicillin (Wako, Japan).

### 2.2 Agr-QS suppressor screening

A luciferin-based, high-throughput assay was developed to screen 577 compounds extracted from natural sources in our laboratory (Arai et al., 2021). Among the 577 compounds screened, 499 were isolated from herbal medicines and 79 were originally from microorganisms. Overnight cultures of the *agr*:P3-*luc* strain were diluted 1:100 into TSB and incubated at 37°C for 4 h with shaking. Cultures were diluted with phosphate-buffered saline and transferred to a 96-well plate (Corning, USA) to obtain a final bacterial concentration of 2 × 10^7^ CFU/well. In experiments investigating the competitive effect between AIP-1 and compounds, 500 *μ*g/L AIP-1 was added. Dimethyl sulfoxide (DMSO, 1%) was added as a control. The culture was incubated at 37°C for 24 h without shaking after which luminescence and an optical density at 600 nm (OD_600_) were measured using Spark (TECAN, Switzerland).

### 2.3 Real-time quantitative PCR

Cultures were grown overnight growth and then diluted 1:1000 in TSB with 100 *μ*M compound or 1% DMSO and incubated at 37°C for 8 h in a static state. The culture was then added to an equal volume of buffer (62.5 *μ*M ethylenediaminetetraacetic acid, 100 *μ*M NaCl, 1% sodium dodecyl sulfate) on a heat block at 95°C and heated for 2 min. Then, the mixture was reacted with an equal volume of phenol: chloroform: isoamyl alcohol (125:24:1) (Sigma, USA) and heated at 95°C for 5 min. The mixture was centrifuged, and the supernatant was used as the lysis sample. RNA was extracted using the Direct-zol RNA Miniprep kit (Zymo, USA), and cDNA was prepared using a PrimeScript™ RT Master Mix (Takara Bio, Japan). Quantitative PCR was performed using Brilliant III Ultra-Fast SYBR^®^ Green QPCR Master Mixes (Agilent, USA) and AriaMx Real-Time PCR System (Agilent, USA). The sequence of primers used for qPCR is listed in Supplementary Table S1; gyrB-F and gyrB-R for *gyrB* (Seidl et al., 2011), RNAIII-F and RNAIII-R for *RNAIII* (Seidl et al., 2011), agrA-F and agrA-R for *agrA* (Wang et al., 2021), hla-F and hla-R for *hla* (Jiang et al., 2023), and psma-F and psma-R for *psm* *α* (Jiang et al., 2018). Gene expression was normalized against that of *gyrB*, and relative expression was calculated using the 2^−ΔΔCT^ method (Livak and Schmittgen, 2001).

### 2.4 Hemolytic toxin assay

The hemolytic toxin assay was performed as previously described, with modifications (Bernheimer, 1988). Briefly, overnight cultures were diluted 1:1000 and incubated in TSB at 37°C with 100 *μ*M compound or 1% DMSO for 4, 8, and 24 h. The cultures were centrifuged, and two-fold serial dilutions of the supernatant were prepared. Rabbit red blood cell solution (Japan BioSerum, Japan) was adjusted to provide an absorbance of 0.80 at 600 nm (A_600_) when completely lysed with the addition of 2.5 mg/mL saponin. Two-fold serial dilutions of the supernatant and rabbit red cell solutions were incubated in a 96-well plate at 37°C for 30 min and centrifuged at 2000 *g* for 3 min. The degree of cell lysis was determined by measuring the A_600_ of the supernatant. Data were fitted to a four-parameter logistic curve and expressed as HA_50_ through nonlinear regression analysis, indicating the inverse of the dilution required for 50% complete lysis.

### 2.5 Growth inhibition assay

Cultures grown overnight were diluted 1:100 in Mueller Hinton Broth (MHB; BD Biosciences, USA) and incubated at 37°C with shaking until an OD_600_ of approximately 0.5 was obtained. Following dilution with MHB, cultures were placed in a 96-well plate with compounds diluted in a two-fold series. This resulted in a final bacterial density of 2.5 × 10^4^ CFU/well. The culture was incubated at 37°C for 24 h without shaking, and bacterial growth was assessed by measuring the OD_620_ using Infinite^®^ 200 PRO (TECAN, Switzerland).

### 2.6 Expression and purification of N-terminally his-tagged AgrAc protein

Plasmid pTKY1297, encoding N-terminally histidine-tagged *agrAc*, was constructed by PCR amplification of a 313-bp BamHI–HindIII fragment carrying *agrAc* from the LAC strain using the primers BamHI-AgrA-F and HindIII-AgrA-R (Supplementary Table S1). The fragment was then cloned into the pUHE212-1 vector (Gamer et al., 1992). To purify N-terminally His-tagged AgrAc, 0.5 L of an *E. coli* DH5*α*Z1 derivative culture harboring pTKY1297 culture was incubated at 37°C for 2 h, and then isopropyl *β*-D-1-thiogalactopyranoside was added to obtain a final concentration of 1 mM for 3 h. The cells were collected by centrifugation and frozen at −80°C. Then, cells were suspended in buffer A (50 mM Tris-HCl pH 7.5, 300 mM NaCl, 10% glycerol, 0.1% Triton X-100) containing 10 mg/mL lysozyme (Sigma, USA) and sonicated on ice. The disrupted cells were centrifuged and the supernatant was incubated with Ni^2+^-NTA agarose (Qiagen, Germany) for 30 min. The AgrAc fusion protein was eluted using buffer A containing 500 mM imidazole. The protein solution was then dialyzed in buffer B (50 mM Tris-HCl pH 7.0, 50 mM NaCl, 1 mM dithiothreitol, 10% glycerol, 0.1% Triton X-100) using a Slide-A-Lyzer Dialysis Cassette (Thermo Scientific, USA) for 2 h. After replacing buffer B, the solution was dialyzed overnight, and the remaining solution was used as the purified AgrAc fusion protein.

### 2.7 Electrophoretic mobility shift assay

To investigate the *in vitro* binding of AgrAc protein to DNA, an electrophoretic mobility shift assay (EMSA) was performed as previously described, with modifications (Sun et al., 2012). Briefly, 100- and 19-bp DNA fragments containing the P3 promoter and LytTR domain were synthesized and the 5’ end was labeled with Cy5 (Eurofins, Japan). DNA fragments were obtained by incubating with oligomer sets, Cy5-agrP3-F and Cy5-agrP3-R, and Cy5-LytTR-F and Cy5-LytTR-R, at 95°C for 5 min. The sequence of oligomers is given in Supplementary Table S1. Physalin H was added at concentrations of 31.25, 62.5, 125, 250, and 500 *μ*M. Other physalins were added at a concentration of 500 *μ*M. Then, 140 nM purified AgrAc fusion protein was added to 10 *μ*L buffer (10 mM Tris-HCl pH 7.4, 50 mM KCl, 5 mM MgCl_2_, 20% glycerol) and incubated at 25°C for 20 min. The mixture was incubated for a further 20 min after adding 100 nM of the DNA fragment. The reaction mixture was subjected to electrophoresis on a native 8% polyacrylamide gel in 0.5× TBE buffer. Labeled DNAs were detected using a Typhoon FLA9000 Photoimager (GE Healthcare, USA).

### 2.8 *In silico* docking analysis to AgrAc

*In silico* docking simulations were performed using AutoDock Vina (Trott and Olson, 2010). Three-dimensional (3D) structures of compounds were prepared using Avogadro (Hanwell et al., 2012) and docked to the B chain of the crystal structure of AgrAc, which is deposited in the Protein Data Bank (PDB) under the accession number 4G4K (Leonard et al., 2012). The grid box was restricted to the DNA-binding region of AgrAc. Since initial simulations suggested that the compound binds to a pocket consisting of H169, N185, and N201, additional calculations were performed with a modified grid box and more flexible side chains near the ligand.

### 2.9 Molecular dynamics study

Molecular dynamics (MD) simulations of AgrA and compounds utilized the Desmond simulation package within the Schrödinger suite 2020-4 (Bowers et al., 2006). Before simulations, AgrA was prepared using the Protein Preparation Wizard in Desmond. An orthorhombic water box was established using the single-point charge (SPC) model (Zielkiewicz, 2006). Counterions were added to neutralize the solvent system, displacing water molecules and equalizing the net charge of the system to minimize its energy. Upon relaxation of the model system, simulations were initiated and performed at 300 K in an NPT (constant particle number, pressure, and temperature) ensemble. The simulations were conducted over 100 ns, with a recording interval of 20 ps for total energy. Interactions between compounds and AgrA were analyzed utilizing Desmond’s Simulation Interaction Diagram. The root mean square derivation (RMSD) for AgrA in the presence of compounds was calculated to examine the structural stability and conformational changes of the AgrA–physalin complexes. Root means square fluctuation (RMSF) analysis was performed to evaluate the conformational and local modifications resulting in physalin binding produced in the amino acid residues of AgrA.

### 2.10 Statistical analysis

Data were statistically analyzed using python 3.7. Data were analyzed using the two-tailed Student’s t-test. ns is used to denote values that are not significantly different.

## 3 Results

### 3.1 Identification of physalin H as an Agr-QS suppressor

**FIGURE 1:**
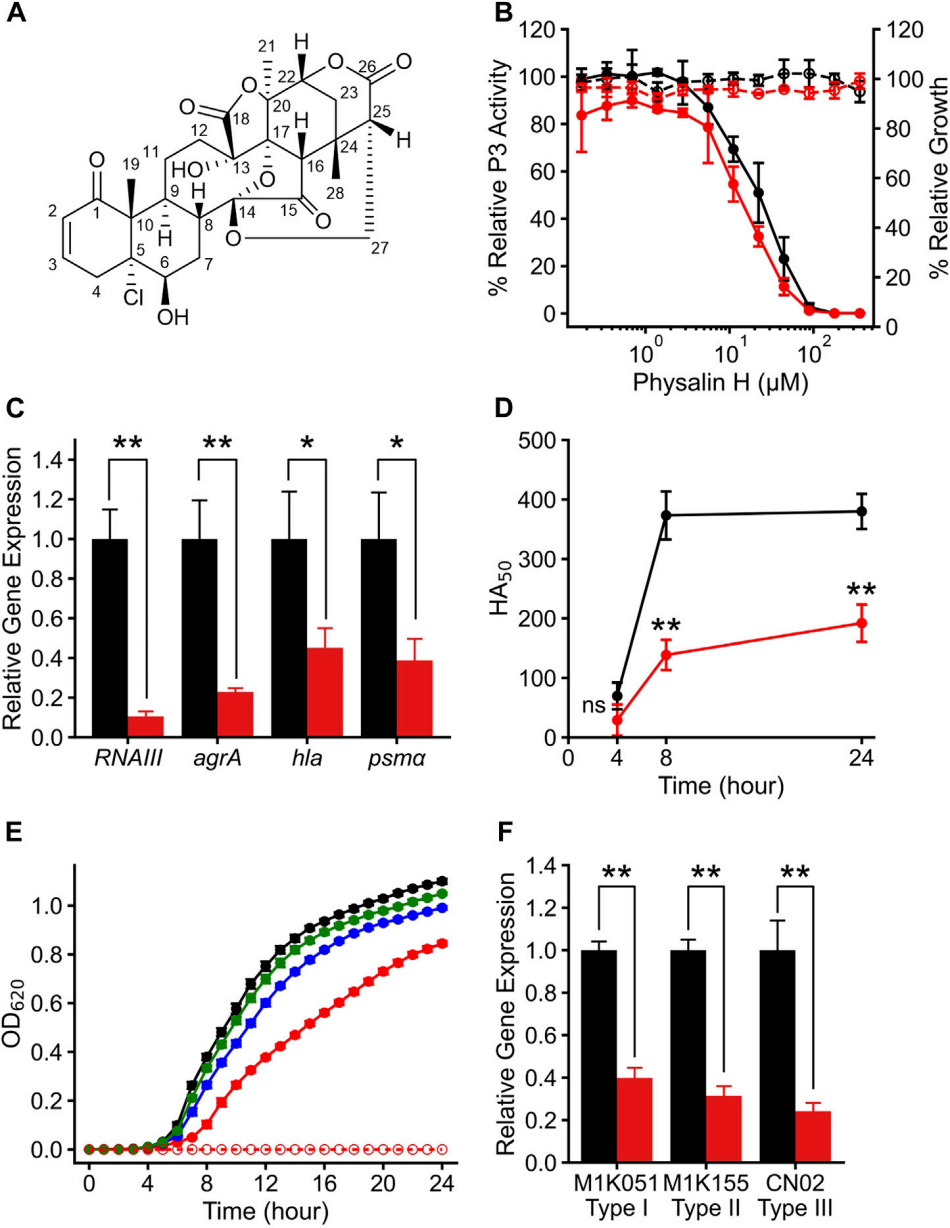
Physalin H suppresses Agr-QS function regardless of the subgroups. **(A)** The structure of physalin H. **(B)** Relative P3 promoter activity (solid line) and relative growth (dot line) when cultured for 24 h without (black) and with (red) autoinducer peptide (AIP) added to media containing serial concentrations of physalin H. P3 promoter activity is expressed relative to that in culture with 1% DMSO, considered as 100% (n = 3). Data are presented as the mean ± standard deviation (SD). **(C)** Normalized *RNAIII*, *agrA*, *hla*, and *psm* *α* expression during 8-h culture of LAC with 100 *μ*M physalin H (red) and 1% DMSO (black) (n = 3). Data are presented as the mean ± SD. *p < 0.05, **p < 0.01, unpaired *t*-test. **(D)** Activity values for 50% hemolyzing red blood cells in culture supernatants of LAC cultured for 4, 8, and 24 h with 100 *μ*M physalin H (red) and 1% DMSO (black) (n = 3). Data are presented as the mean. **p < 0.01, ns, not significantly different, unpaired *t*-test. **(E)** Growth curve for 24-h culture of LAC strain with 50 (green), 100 (blue), and 200 (red) *μ*M physalin H and 1% DMSO (black). OD_620_ of 24-h medium with 200 *μ*M physalin H is indicated as a control medium containing physalin H (dot line). **(F)** Normalized *RNAIII* expression in the 8-h culture of Agr-I strain M1K051, Agr-I I strain M1K155, and Agr-III strain CN02 with 100 *μ*M physalin H (red) and 1% DMSO (black) (n = 3). **p < 0.01, unpaired *t*-test.

*S. aureus* strain LAC, the epidemic methicillin-resistant USA300 clone, Agr-QS subgroup I, the predominant subgroup among all subgroups of Agr-QS, has been widely studied (Wang et al., 2007). First, we screened 577 compounds that inhibit *agr*:P3 activation using a reporter strain from *S. aureus* LAC, in which P3 drives the production of luminescence (Nakamura et al., 2013). To identify compounds that could inhibit Agr-QS expression without repressing bacterial growth, 100 *μ*M of each compound was added to 2 × 10^7^ CFU/mL of bacteria and cultured for 24 h (Supplementary Figure S1A). Although 85 compounds generated luminescence levels less than 1% compared to those obtained with 1% DMSO, 81 compounds were considered to induce growth inhibition because they resulted in an OD_600_ of 90% or lower than that obtained with DMSO (Supplementary Figure S1B). Overall, four compounds generated OD_600_ of >90%. The four compounds were physalin H isolated from *Solanum nigrum* (Arai et al., 2014) (Figure 1A), lasidiol *p*-methoxybenzoate isolated from *Xanthium strumarium* (Karmakar et al., 2015), ovatodiolide isolated from *Hyptis suaveolens* (Arai et al., 2015), and ceanothic acid isolated from *Zizyphus cambodiana* (Arai et al., 2008), suggesting that these compounds can suppress Agr-QS in MRSA without repressing bacterial growth.

To assess the specificity of Agr-QS suppression by physalin H, *agr*:P3 activity was examined in the presence of serial concentrations of physalin H (Figure 1B). Regardless of the concentration of physalin H, the OD_600_ and CFU values of culture after 24 h were comparable to those with 1% DMSO (Supplementary Figure S2A). A gradual concentration-dependent decrease in *agr*:P3 activity was observed with the addition of physalin H at concentrations of 5 *μ*M and above, and no activity was observed when physalin H was added at 100 *μ*M and above. Based on this, the IC_50_ was calculated as 20.4 *μ*M. Agr-QS activity increases during the exponential growth phase, inducing the expression of *RNAIII* and *agrA* (Novick et al., 1993). There was a marked decrease in the expression of *RNAIII* and *agrA* in LAC cells following 8 h culture with 100 *μ*M physalin H (Figure 1C). RNAIII activates the expression of *hla*, encoding the hemolytic toxin, and phosphorylated AgrA activates the expression of *psm* *α* and *psm* *β*, encoding other hemolytic toxins (Queck et al., 2008). Additionally, the expression of *hla* and *RNAIII* decreased following 8 h culture of LAC cells with 100 *μ*M physalin H (Figure 1C). Hemolytic toxins, such as Hla and phenol-soluble modulins (PSMs), are secreted by bacterial cells (Patel1 and Rawat2 (2023)). To examine the effect of physalin H on toxin production, hemolytic activity in the supernatant from LAC cells was determined (Figure 1D). Hemolytic activity in supernatants was repressed after 4, 8, and 24 h culture with physalin H compared with that in supernatants from culture with DMSO. Physalin H has also been reported to possess leishmanicidal activity (Choudhary et al., 2006). When physalin H was added to cultures with a bacterial count of ∼ 2.5 × 10^5^ CFU/mL and incubated for 24 h, high concentrations (200 *μ*M) slightly inhibited bacterial growth (Figure 1E; Supplementary Figure S2B). These results indicated that physalin H inhibits Agr-QS expression in MRSA, suppressing the production of virulence factors.

To elucidate the target of physalin H in Agr-QS suppression, AIP-1 was added and *agr*:P3 activity was determined (Figure 1B). The addition of AIP-1 had no effect on the physalin H–dependent inhibition of P3 activity (IC_50_ = 17.1 *μ*M). Furthermore, reduced *RNAIII* expression level was observed in strains other than LAC, including M1K051 (Agr-QS subgroup I) and M1K155 (Agr-QS subgroup II) isolated from human skin, and CN02 (Agr-QS subgroup III) isolated as a nosocomial infection when cultured in the presence of 100 *μ*M physalin H (Figure 1F). These results suggested that physalin H suppresses the function of AgrA in *S. aureus* rather than inhibiting the interaction between AIP and AgrC.

### 3.2 Effect of physalin structure on Agr-QS suppression

**FIGURE 2:**
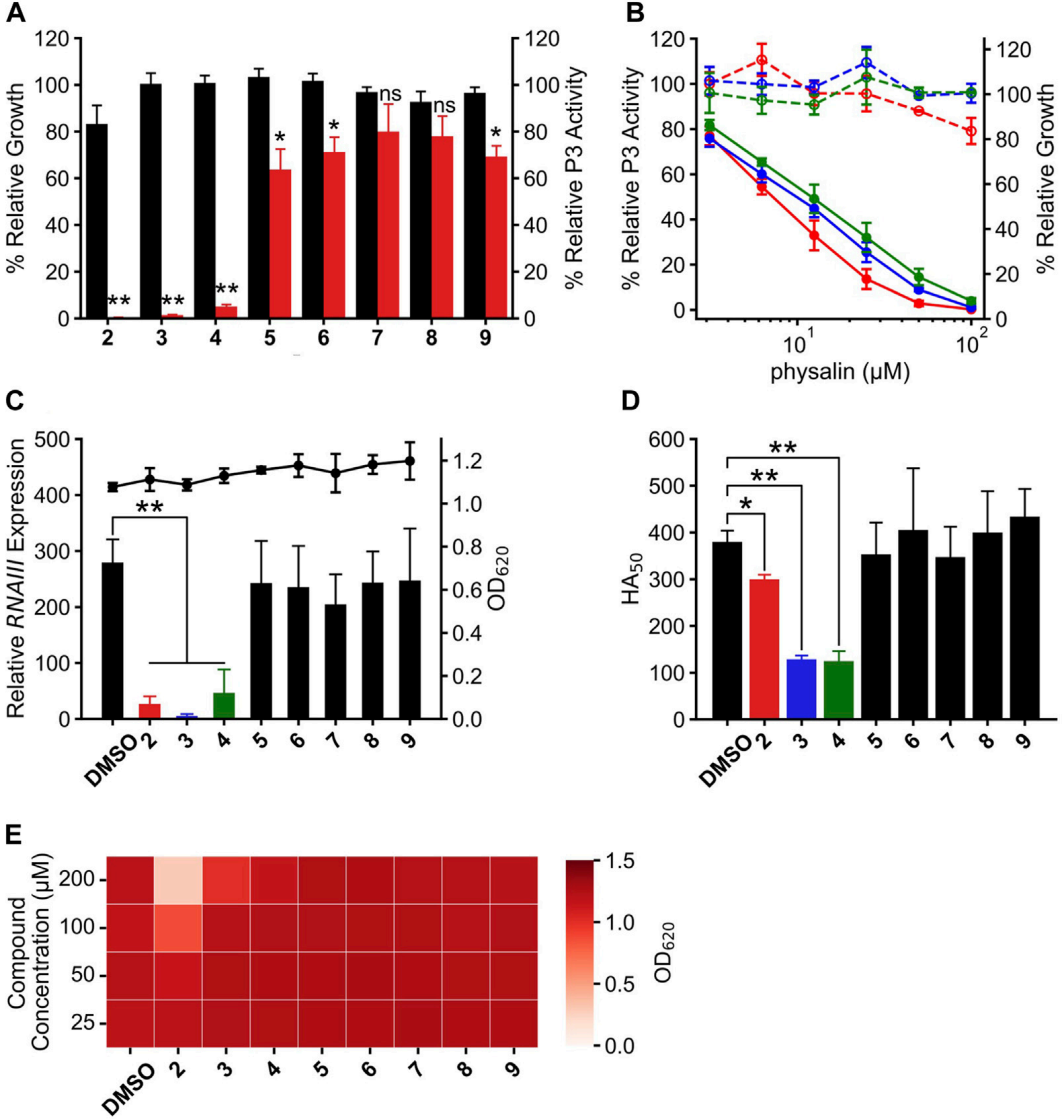
Physalin F, physalin B, and isophysalin B can suppress Agr-QS expression. **(A)** Relative P3 promoter activity and growth during 24-h culture with 100 *μ*M physalins (PS) two to nine% and 1% DMSO (n = 3). Data are presented as the mean ± SD. *p < 0.05, **p < 0.01, ns, not significantly different, unpaired *t*-test. **(B)** Relative P3 promoter activity (solid line) and relative growth (dot line) during 24-h culture with serial concentrations of PS2 (physalin F, red), PS3 (physalin B, blue), and PS4 (isophysalin B, green). P3 promoter activity is expressed relative to activity in cultures without PSs, considered as 100% (n = 3). Data are presented as the mean ± SD. **(C)** Normalized *RNAIII* expression (bar) and growth (line) during 8-h culture of LAC with 50 *μ*M PS2, 100 *μ*M PSs3–9 and 1% DMSO (n = 3). Data are presented as the mean ± SD. **p < 0.01, unpaired *t*-test. **(D)** Activity values for 50% hemolyzing red blood cells in culture supernatants of LAC cultured for 24 h with 50 *μ*M PS2, 100 *μ*M PSs3–9 and 1% DMSO (n = 3). Data are presented as the mean ± SD. *p < 0.05, **p < 0.01, unpaired *t*-test. **(E)** OD_620_ after 24-h culture with LAC strain at 2.5×10^5^ CFU/mL with serial concentrations of PSs2–9 and DMSO.

In addition to physalin H, eight physalins, PSs2–9, with different structures were isolated from *P. minima*; there were no significant differences in the physicochemical properties of these physalins (Supplementary Figure S3 **;** Supplementary Table S2). These physalins are composed of eight rings, A–H, and only a small part of the difference in the structures between these physalins was in their A and B rings (Hosoya et al., 2008; Manome et al., 2023). The effect of PSs2–9 on Agr-QS suppression was investigated to clarify which substitution of the physalin structure is required for Agr-QS inhibition. Each physalin (100 *μ*M) was added and the growth rate and *agr*:P3 activity were examined after 24 h (Figure 2A). Consequently, *agr*:P3 activity was almost completely absent in cells treated with PSs2 (physalin F), 3 (physalin B), and four (isophysalin B); however, *agr*:P3 activity remained at around 49%–61% with PSs5–9. When 100 *μ*M PS2 was added, proliferation was about 80% compared with when 1% DMSO was added. However, like physalin H, treatment with PS2 also decreased *agr*:P3 activity in a concentration-dependent manner, even at 50 *μ*M, which did not repress growth (IC_50_ = 7.22 *μ*M). A concentration-dependent decrease in *agr*:P3 activity was also observed following treatment with PSs3 and 4, with IC_50_ values of 9.22 and 11.6 *μ*M, respectively (Figure 2B). To confirm the effect of PSs2–9 on *RNAIII* expression in LAC cells, we examined *RNAIII* expression in LAC cells cultured for 8 h with 50 *μ*M PS2 or 100 *μ*M PSs3–9. The degree of *RNAIII* expression with PSs2, 3, and four was significantly decreased, whereas the degree of *RNAIII* expression with PSs5–9 was the same as that with DMSO (Figure 2C). The hemolytic activity of the 24-h culture supernatant following treatment with PSs3 and four was significantly reduced; however, that of culture supernatants following treatment with other physalins, including PS2, was similar to that of the culture with DMSO (Figure 2D). These results suggested that PSs3 and 4, in addition to physalin H, could repress virulence by inhibiting Agr-QS expression. Physalin D possesses antimicrobial activity against *S. aureus* with an MIC of 32 *μ*g/mL (62.7 *μ*M) (Helvaci et al., 2010). However, the MIC of PS5, corresponding to physalin D, against LAC exceeded 200 *μ*M (Figure 2E). With PS2, weak growth inhibition was observed at 100 *μ*M, and strong growth inhibition was observed at 200 *μ*M. With PS3, weak growth inhibition was observed at 200 *μ*M. No growth inhibition was observed with other PSs, even when 200 *μ*M was added (Figure 2E).

### 3.3 Inhibition of AgrA protein bound to P3 promoter by physalin H

**FIGURE 3:**
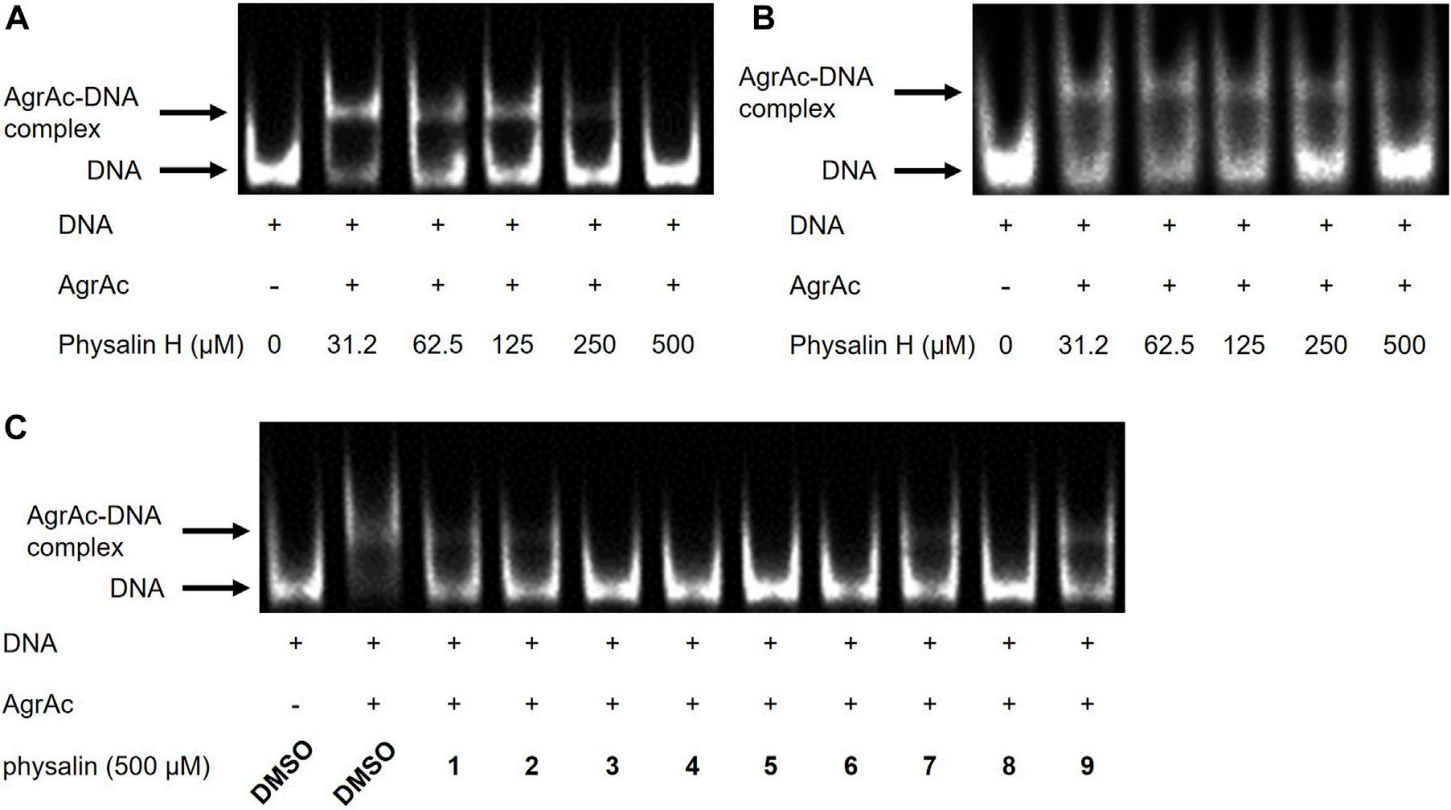
Physalins inhibit the binding of AgrAc to DNA. **(A) (B)** Effect of increasing concentrations of physalin H (31.25–500 *μ*M) on His^6^-AgrAc–Cy5-labeled 100-bp **(A)** and 19-bp **(B)** oligonucleotide complex formation by electrophoretic mobility shift assay (EMSA). **(C)** Effect of 500 *μ*M PSs1–9, on AgrAc–Cy5-labeled 100-bp oligonucleotide complex formation by EMSA.

AgrA binds to the P2 and P3 regions in the *agr* locus via its C-terminal DNA-binding domain (residues 138–238), termed AgrAc (Sidote et al., 2008). There is a high-affinity LytTR domain–binding site and a low-affinity binding site in the P3 region (Koenig et al., 2004). Therefore, to clarify whether physalin H inhibits the binding of AgrA to the P3 region, the effect of physalin H on the *in vitro* interaction between AgrAc and P3 DNA was investigated via EMSA (Figure 3A). The P3 region contains two binding sites for AgrAc; therefore, incubating purified N-terminally histidine-tagged AgrAc (His^6^-AgrAc) with a 100-bp oligonucleotide of the P3 region shifted the electrophoretic migration of Cy5-labeled nucleotides. DNA migration gradually decreased as the concentration of physalin H increased, and no DNA migration was observed following the addition of 500 *μ*M physalin H. A 19-bp oligonucleotide containing the 9-bp consensus LytTR-binding sequence flanked by five bp on each side is sufficient for a stable interaction between AgrAc and DNA oligonucleotide (Sidote et al., 2008). DNA migration also shifted when 19-bp Cy5-labelled oligonucleotide and His^6^-AgrAc were mixed; however, this was lost following the addition of 500 *μ*M physalin H (Figure 3B). Thus, physalin H blocks the stable interaction between AgrA and *agr* promoter regions.

### 3.4 Docking state between AgrAc and physalins contributes to Agr-QS suppression

To investigate whether differences in the Agr-QS inhibitory activity of physalins were due to differences in the inhibition of the AgrA–DNA interaction, we examined the binding between His^6^-AgrAc and 100-bp oligonucleotide with the addition of 500 *μ*M of each physalin. All physalins inhibited the binding of AgrAc to DNA *in vitro* (Figure 3C).

**FIGURE 4:**
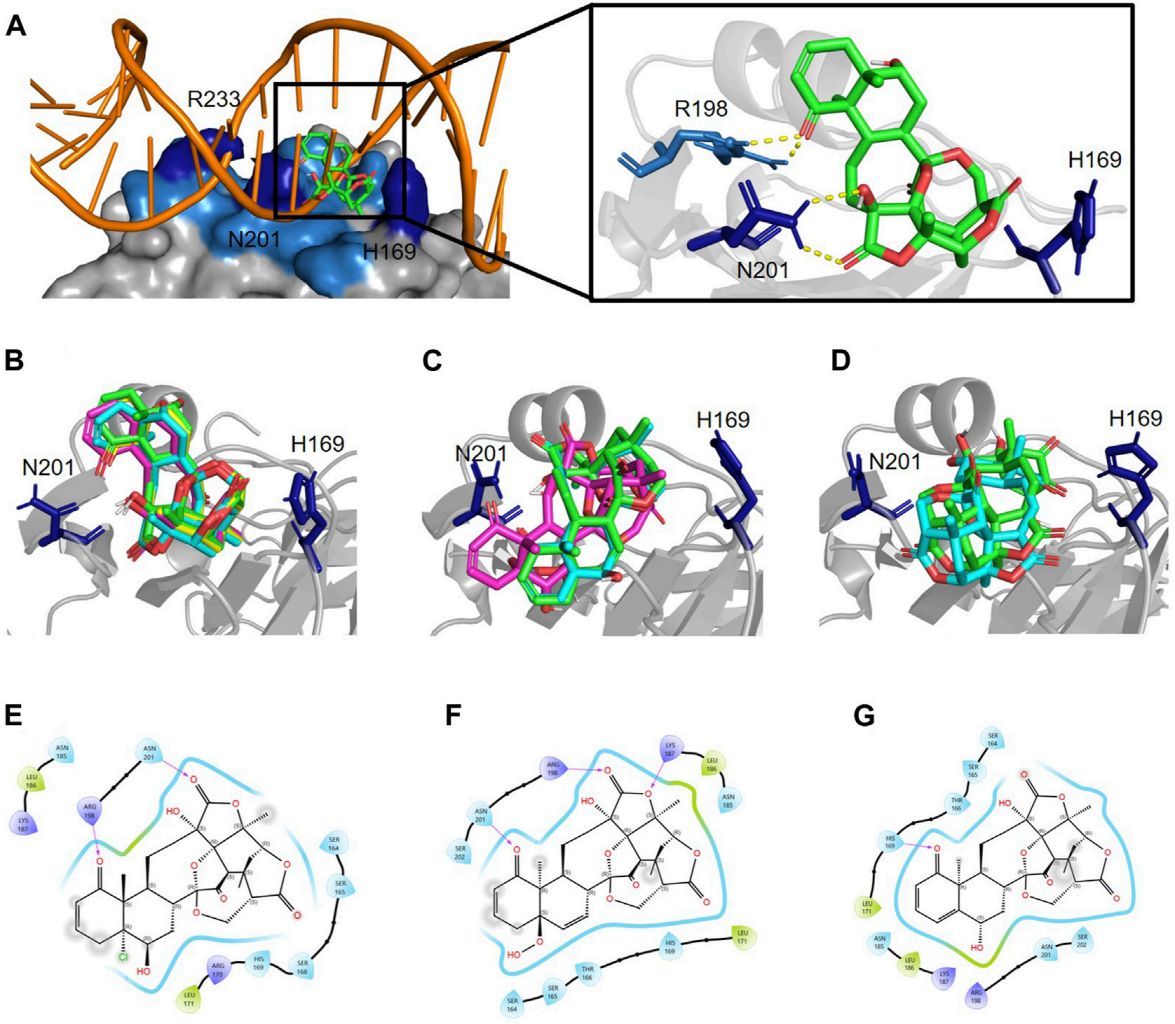
Physalins that suppress Agr-QS interact in a different state of interaction with the site of AgrA from physalins without the ability of Agr-QS suppression. **(A)** *In silico* docking of physalin H to AgrAc from *Staphylococcus aureus* (PDB ID: 4G4K). Space-filled representation of the C-terminal AgrA binding domain (blue) bound to target DNA. Surface residues that directly interact with DNA are shown in dark blue. Physalin H is represented as a stick. An enlarged view of the boxed area shows the physalin H-docking site and surrounding residues. The dotted line indicates H-bound between ketone structure at C-1 and C-18 of physalin H, and R198 and N201 of AgrA. **(B)** Docking state A of four physalins with Agr-QS-suppression activity. Physalin H (green), PS2 (cyan), PS3 (magenta), and PS4 (yellow). **(C)** Two-dimensional (2D) interaction of AgrA and physalin H. **(D)** Docking state B of three physalins without Agr-QS-suppression activity. PS5 (green), PS6 (cyan), and PS7 (magenta). **(E)** 2D interaction of AgrA and PS5. **(F)** Docking state C of two physalins without Agr-QS-suppression activity. PS8 (green) and PS9 (cyan). **(G)** 2D interaction of AgrA and PS8.

The 9-bp consensus sequence interacts with AgrAc, and several bases make specific contacts with residues H169, N201, and R233 in AgrAc. The region flanking the 9-bp consensus sequence is involved in nonspecific DNA backbone interactions with AgrAc and appears to contribute to stability rather than specificity (Sidote et al., 2008). An *in silico* docking analysis between AgrAc and physalins (Figure 4) revealed three states where physalins interact, although the docking scores were similar, ranging between −10.3 and −8.9 kcal/mol (Table 1). Physalin H, with a docking score of −10.3 kcal/mol, formed a hydrogen bond with N201 of AgrAc through the carbonyl oxygen at C-18 in the E ring, and with R198 through the carbonyl oxygen at C-1 in the A ring (Figures 4A–C). Moreover, the hydroxyl group at C-13 may also interact with R198 through a hydrogen bond. Although the atoms used for hydrogen bonding between physalin H and AgrAc are common to all physalins, the entire ring structure of PSs2–4 interacted with AgrAc at the same location and in the same direction as physalin H (Figure 4B). However, the sites in PSs2–4 and the residues in AgrAc that form hydrogen bonds differed from those for physalin H (Table 1). The interaction of physalin H and PSs2–4 with AgrAc was designated as docking state A.

**TABLE 1:** The molecular docking results between AgrAc (PDB ID: 4G4K) and physalins

Docking state	PS	Compound name	Docking score	H-bond
			(kcal/mol)	interaction
A	1	Physalin H	−10.3	R198, N201
	2	Physalin F	−9.7	R198
	3	Physalin B	−9.7	S202
	4	Isophysalin B	−10.0	N185
B	5	5*α*-hydroperoxy-6,7-	−9.3	K187, R198, N201
		didehydro-5,6-		
		dihydrophysalin B		
	6	Physalin K	−9.5	R198, N201
	7	Physalin D	−9.4	–
C	8	Physalin XII	−8.9	H169, N185, K187
	9	Physalin G	−9.5	H169

To enable PSs5 and six to interact with AgrAc, termed docking state B, PSs5 and six were placed in a similar position to that of physalins in docking state A by forming a hydrogen bond with R198 and N201 of AgrAc (the same as that formed by physalin H). However, the direction of the ring was opposite to that with physalins in docking state A because PSs5 and six form a hydrogen bond with N201 of AgrAc through the carbonyl oxygen at C-1, and with R198 through the carbonyl oxygen at C-18 (Figures 4D,E; Table 1). The docking simulation between PS7 and AgrAc suggested that PS7 located the docking state B position, although it did not form hydrogen bonds with any residues of AgrAc. PSs8 and nine interacted with AgrAc, termed docking state C, at a different position to that of physalins in docking states A and B. Furthermore, hydrogen bonds formed with the amino acid residues of AgrAc also differed (Figures 4F,G; Table 1). The carbonyl oxygen at C-1 in PSs8 and nine formed a hydrogen bond with H169 of AgrAc (Figure 4G). This may be due to differences in the binding state with AgrA; although Agr-QS inhibition was observed in physalins in docking state A, there was minimal Agr-QS inhibition for physalins in docking states B and C.

### 3.5 Agr-QS suppression due to the stable interaction between L186 of AgrA and the carboxyl oxygen at C-15 of physalins

**FIGURE 5:**
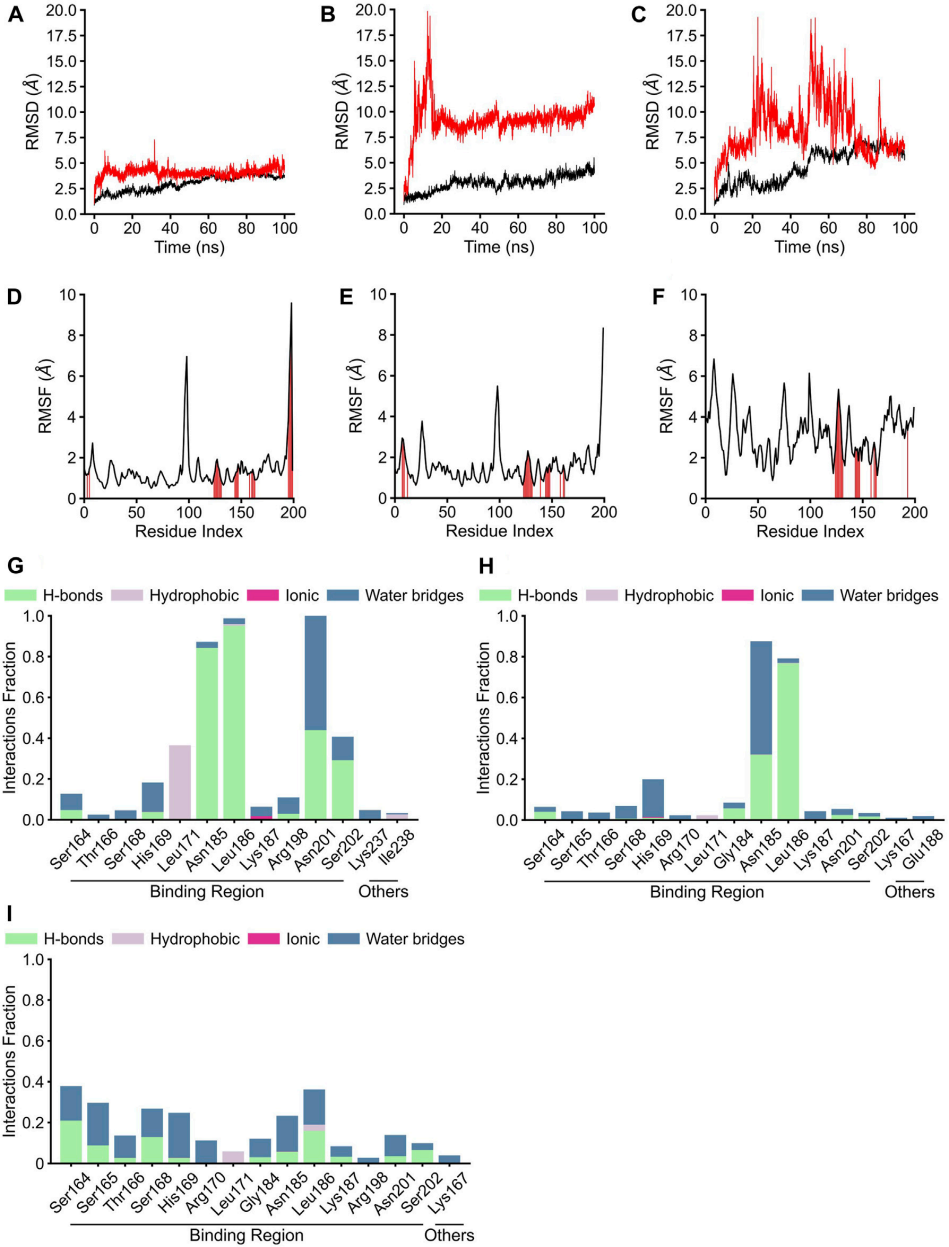
Molecular dynamics analysis of three physalin conformations bound to AgrA. **(A–C)** The results of protein root mean square derivation (RMSD) (black line) and ligand RMSD (red line). **(D–F)** The root mean square fluctuation (RMSF) plot of residue index. The red position indicates the residue index of AgrA bound by the ligand. **(G–I)** The residues of AgrA interacted with physalins. The results of RMSD, RMSF, and interaction of physalin H **(A**, **D**, **G)**, PS5 **(B**, **E**, **H)**, and PS8 **(C**, **F**, **I)** bound to AgrA.

Although the positions of hydrogen bonds formed between the amino acid residues of AgrA and physalins in docked states A, B, and C differed, in each state, physalin interacted with amino acid residues important for AgrA binding with DNA (Figure 4; Table 1). To investigate the stability of the interaction between AgrA and physalins in docking states A, B, and C, 100-ns MD simulations were performed with AgrA and physalin H, PS5, and PS9 (Figure 5). First, we calculated the RMSD in the presence of physalins (Figures 5A–C). The protein RMSD of the AgrA–physalin H complex fluctuated up to around 2.0 Å for 10 ns and then to around 3.8 Å, with an average value of 2.96 Å. The ligand RMSD value also fluctuated up to 10 ns and then stabilized around 5.0 Å with an average value of 4.09 Å. This similar RMSD pattern suggested that physalin H stably binds to the binding pocket of AgrA. In contrast, the protein RMSD for AgrA–PS5 was similar to that obtained for physalin H; however, the ligand RMSD fluctuated up to 15 ns and then stabilized around 9.5 Å. The average values obtained for protein and ligand were 3.02 and 9.17 Å, respectively, indicating that the stability of PS5 in the docking pocket of AgrA was less than that of physalin H. For AgrA–PS9, the ligand RMSD was unstable and highly variable with an average value of 8.28 Å, and the protein RMSD stabilized at approximately 7 Å after 50 ns with an average value of 4.66 Å.

The RMSF plots for physalin H and PS5 were highly similar, with average RMSF values of 1.39 and 1.58 Å, respectively (Figures 5D,E). In the binding between AgrA and both physalins, although the residue index around 90–100 fluctuated, this position coincided with the boundary between the N- and C-terminal domains of AgrA. The AgrA region bound by both physalins was concentrated at a residue index around 130–150. In the binding between AgrA and PS9, although the binding sites of physalin were gathered in the same residue index, the PMSF plots were not stable compared with those for the other two physalins with an average RMSF value of 3.17 Å (Figure 5F).

AgrA–physalin interactions were monitored throughout the 100-ns simulation. As shown in Figure 5G, physalin H interacted with residues N185, L186, and N201 for >90% of the simulation period. Among interactions with N185 and L186, hydrogen bonds were maintained for more than 95% of the simulation period, and water bridges and hydrophobic contacts appeared partially. The interaction with N201 was present for about 40% of the simulation period through a hydrogen bond and for about 60% of the simulation period through a water bridge. The S202 residue interacted for about 40% of the simulation period, and of the 40%, 80% was through a hydrogen bond and the rest was through a water bridge. The L171 residue interacted for about 40% of the simulation period, and this interaction was a hydrophobic contact. Other than L237 and I238, the interacting residues were located in the DNA-binding region. In contrast, the number and position of amino acid residues in the DNA-binding region on AgrA with which the three physalins interacted were highly similar; however, unlike with physalin H, no residues continued to interact with PSs5 and nine in the 100-ns simulation, except for the interactions of PS5 with N185 and L186 (Figures 5H, I). The interaction of PS5 with L186 maintained a hydrogen bond for about 80% of the simulation period. The interaction between PS5 and N185 was present for about 90% of the simulation period, of which 30% was through hydrogen bonds and the rest through water bridge. These results suggest that the interaction between physalin H and AgrA is more stable than that between PSs5 and 9.

**FIGURE 6:**
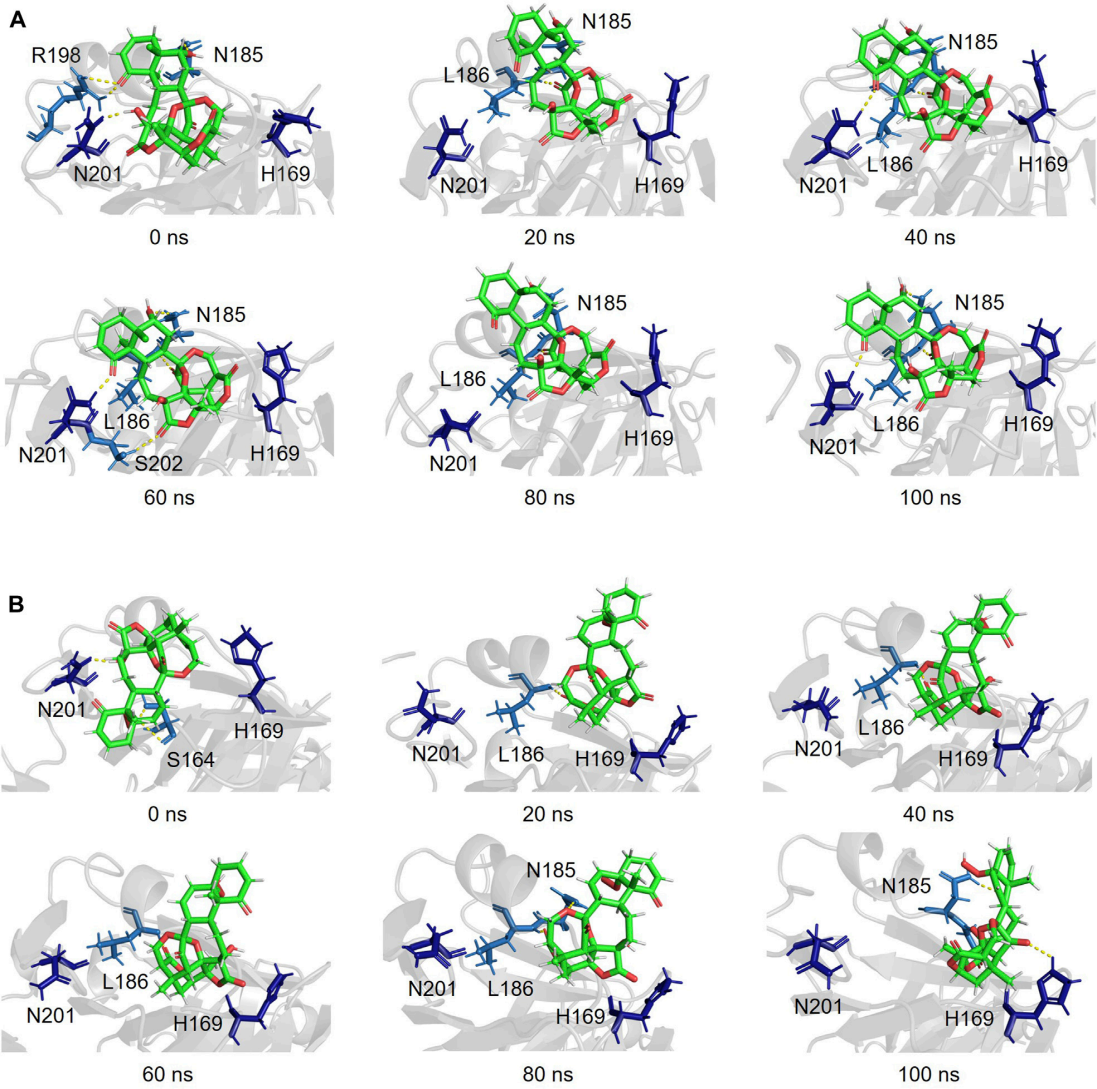
Simulated complex of the DNA binding region of AgrA in the presence of physalin H **(A)** and PS5 **(B)** at 0, 20, 40, 60, 80, and 100 ns.

Snapshots of AgrA–physalin H every 20 ns in the 100-ns simulation revealed how the interaction between physalin H and the amino acid residues of AgrA changes **(** Figure 6A; Table 2). At 0 ns, hydrogen bonds were formed between R198 of AgrA and the carbonyl oxygen at C-1 of physalin H, and between N201 of AgrA and the hydroxyl group at C-13 of physalin H, similar to the results obtained in the *in silico* docking simulation (Figure 4A). In addition, the hydroxyl group at C-6 of the B ring in physalin H interacted with N185 of AgrA through a hydrogen bond. At 20 ns, the two former hydrogen bonds were lost; however, the hydrogen bond between N185 and the hydroxyl group at C-6 remained, and an additional hydrogen bond was formed between L186 of AgrA and the carbonyl oxygen at C-15 of physalin H. Hydrogen bonds between N185 and the hydroxyl group at C-6, and between L186 and the carbonyl oxygen at C-15 were observed up to 100 ns. Hydrogen bonds between N201 and the carbonyl oxygen at C-1 were observed at 40, 60, and 100 ns. Furthermore, MD analysis of AgrA with PSs2–4, which have reduced Agr-QS activity in addition to physalin H, suggested that they continued to interact with L186 of AgrA and the carboxyl oxygen at C-15 of physalins through hydrogen bonds in the 100-ns simulation (Table 2; Supplementary Figure S4). In addition, N201 of AgrA interacted with the carboxyl oxygen at C-1 several times during the simulation period. These results suggest that the hydrogen bond formed between L186 and the carbonyl oxygen at C-15 acts as an anchor, sustaining the interaction between N201 and the carbonyl oxygen at C-1 of physalin H, PSs2, 3, and 4. It is possible that these interactions allow physalin to fit stably into the DNA-binding site of AgrA, thereby preventing AgrA from binding to the *agr* promoter. In contrast, PS5 formed a hydrogen bond with L186 at the carbonyl oxygen at C-26 in the G ring for 20–80 ns; however, this interaction did not allow PS5 to remain in the DNA-binding pocket of AgrA. Rather, it caused the entire structure of physalin to be pushed outside (Figure 6B).

**TABLE 2:** Hydrogen bonding and water bridging between oxygen of physalins and amino acid residue of AgrA in the 100-ns simulation.

Position of oxygen in PS		Residue of AgrA: hydrogen bond, water bridge (W)					
		0 ns	20 ns	40 ns	60 ns	80 ns	100 ns
PS1	O at C-1	R198	N201(W)	N201	N201		N201
	OH at C-6	N185	N185	N185	N185	N185	N185
	OH at C-13	N201					
	O at C-15		L186	L186	L186	L186	L186
	O at C-18		S164(W)		S202	N201(W)	
PS2	O at C-1	N201(W)	N201(W)		N201(W)	N201(W)	N201(W)
	O at C-5	N185		N185			
	O at C-6	N185		N185			
	OH at C-13	N201	N201(W)			N201(W)	N201(W)
	O at C-15		L186	L186	L186	L186	L186
	O at C-18	S202(W)		S202(W)		S202(W)	
	O at C-22				H169(W)		
	O at C-26				H169(W)		
PS3	O at C-1	R198		N201(W)			N201(W)
	OH at C-13	N201				N201(W)	
	O at C-14	N185(W)					
	O at C-15	N185(W)	L186	L186	L186	L186	L186
	O at C-18	S202(W)	S202(W)	N201(W)			N201(W)
	O at C-27	N185(W)					
PS4	O at C-1	N201(W)	N201	N201			
	O at C-15	L186(W)	L186	L186	L186	L186	L186
	O at C-18	N201, S202(W)	S202	S202		S202(W)	S202(W)
	O at C-22		S168(W)				
	O at C-26		S168(W)	H169(W)			

## 4 Discussion

In this study, based on screening 577 compounds including 499 isolated from herbal medicines, we could identify physalin H, physalin B, and isophysalin B as novel modulators of Agr-QS of MRSA. Furthermore, we determined AgrA as target of physalins in Agr-QS suppression. In addition, lasidiol *p*-methoxybenzoate, ovatodiolide, and ceanothic acid were identified via screening as suppressors of Agr-QS expression. All compounds that were found to suppress Agr-QS expression were isolated from herbal medicines. Although the number of compounds in our library was not large, we succeeded in identifying several novel Agr-QS modulators. This suggests that plant-derived products may contain more compounds with Agr-QS suppression activity.

Physalins possess a highly oxygenated, complex structure, and are divided into two subgroups: Type I and Type II (Meira et al., 2022). The physalins used in this study were classified as Type I and carried an oxygen heterocyclic bridge between C-14 and C-27. Most physalins reported to date have Type I structures, and the biological activity of physalin B has been well investigated (Wu et al., 2021). Previously, physalin B (200 *μ*g/mL) was shown to inhibit *S. aureus* ATCC6538P, which is an antibiotic-sensitive strain, by 85% using an agar diffusion assay (Silva et al., 2005). In the present study, physalin B (PS3) exerted a slight growth inhibitory effect against the epidemic methicillin-resistant USA300 strain at a concentration of 200 *μ*M (102 *μ*g/mL). Although physalin B can inhibit the growth of *S. aureus* regardless of antibiotic resistance or toxin acquisition, the growth inhibitory activity is less than Agr-QS suppression activity because *agr*:P3 activity was almost completely absent at a concentration of 100 *μ*M. Furthermore, growth inhibition was observed with physalin F (PS2) at 100 *μ*M and above. Physalin F suppressed Agr-QS expression to the same extent as done by physalin B; however, inhibition of hemolytic activity was low. The USA300 strain has acquired various toxins, including hemolytic toxins whose expression is not controlled by Agr (Patel1 and Rawat2 (2023)). It is possible that physalin F suppressed Agr-QS and lysed bacteria due to its cytotoxic effects, by releasing intracellular toxins and weakening anti-hemolytic activity.

Since physalins are characterized by their A and B rings, the side chains of these rings may be involved in interaction with factors targeted by each physalin. For example, the A ring of physalin A could form a covalent bond with cysteine residues of inhibitor of nuclear factor kappa-B kinase subunit *β* (Ji et al., 2012). In the present study, physalins were found to possess carbonyl oxygens at C-1 in the A ring, which is involved in the interaction with AgrA. However, the residues of AgrA that interacted with physalins with Agr-QS inhibitory activity and physalins without Agr-QS inhibitory activity differed. The hydroxyl group at C-6 in the B ring of physalin H could interact with N185 of AgrA through a hydrogen bond. Conversely, whereas PSs6–8 possess the same hydroxyl group, their hydroxyl group at C-6 was unable to form a hydrogen bond with any residues of AgrA. Therefore, the hydrogen bond with AgrA through the hydroxyl group at C-6 of physalin H may function as an aid to the interaction. Differences in the structure of the A and B rings in physalins affect the characteristics of the compounds. Our results suggest that structural differences between the A and B rings could change the position of the oxygen atom that is normally present in each physalin, resulting in a specific interaction with each target protein.

Regulators of bacterial transcription combine a signal input domain with some version of the DNA-binding helix-turn-helix (HTH) domain (Aravind et al., 2005). In contrast, AgrA carries a non-HTH DNA-binding domain, called a LytTR domain (Nikolskaya and Galperin, 2002). AgrA interacts with a 9-bp conserved binding sequence (5′-ACAGTTAAG-3′) in the P3 promoter via its LytTR domain. The interaction of H169 in AgrA with the ninth G through a direct hydrogen bond is essential for DNA binding (Sidote et al., 2008). PSs8 and nine can interact with H169 of AgrA via the carboxy oxygen at C-1; however, this interaction is unstable. The unstable interaction between AgrA and PSs8–9 might result in a slight decrease in P3 activation in *S. aureus* cells. In AgrA, L186 is located where it contacts the consensus sequence on the same DNA strand side as the H169 interaction (Sidote et al., 2008). Although PS5 was found to form a stable interaction with L186 through hydrogen bonding with the carbonyl oxygen of C-26, it did not strongly suppress Agr-QS expression. Thus, Agr-QS expression cannot be weakened even if it stably interacts with L186 of AgrA, which does not directly interact with DNA bases.

Physalins that reduced Agr-QS expression formed a stable hydrogen bond between L186 of AgrA and the carboxyl oxygen at C-15. The interaction between the carbonyl oxygen at C-15 of physalins and L186 of AgrA maintains the carbonyl oxygen at C-1 in physalins where it can interact with N201 of AgrA, thereby preventing AgrA from binding to DNA. N201 of AgrA contacts the consensus sequence through a water bridge in the minor groove, and this alanine substitution also weakens its binding to DNA (Sidote et al., 2008). Although the interaction with the carbonyl oxygen at C-1 involves contact via hydrogen bonding and water bridging, the interaction between AgrA and the consensus sequence of DNA could be strongly suppressed by covering the part that interacts with DNA.

LytTR-containing proteins account for ∼2.7% of all prokaryotic response regulators (Galperin, 2006). Although LytTR domains are typically found in just one or two proteins per genome, they regulate the production of many important virulence factors in bacteria (Galperin, 2008). Differences in the structure of physalins altered the interaction with the consensus sequence of AgrA; therefore, physalins may suppress the activity of species-specific LytTR proteins in bacteria. Next-generation antimicrobials that target bacterial virulence factors to disrupt pathogenic potential without impacting bacterial viability have been studied as alternatives to traditional antibiotics to combat the increasing prevalence of antibiotic-resistant bacteria (Gadar and McCarthy, 2023). Compounds that target the LytTR protein are potential candidates as alternatives to antibiotics. Savirin and azan-7 inhibit Agr-QS function in *S. aureus,* but not AgrA activity in *S. epidermidis,* by binding to a different LytTR domain than that bound by physalins (Sully et al., 2014; Bernabe et al., 2021). Cinnamaldehyde, a natural food preservative, targets the LytTR DNA-binding domain of AgrA to attenuate biofilm formation of *Listeria monocytogenes* (Jiang et al., 2023). Research into compounds that specifically bind to these LytTR proteins will be useful in the development of next-generation antimicrobials.

In conclusion, physalin H, physalin B, and isophysalin B suppress Agr-QS function by inhibiting the interaction between AgrA and the *agr* promoter, regardless of their cytotoxicity. In addition, docking and MD simulations revealed that differences in the structure of physalins do not directly affect the interaction with AgrA but rather contribute to determining the interaction between the carbonyl oxygen of physalins and AgrA. However, physalins suppress Agr-QS function at high concentrations; therefore, it is necessary to achieve this effect at a lower concentration to treat MRSA infection *in vivo*. The information regarding the interaction between AgrA and physalins revealed in this study may help design effective compounds in the future.

## Data Availability

The original contributions presented in the study are included in the article/Supplementary Material, further inquiries can be directed to the corresponding author.
