# Supplementary material for: Physalin H, physalin B, and isophysalin B suppress the quorum-sensing function of Staphylococcus aureus by binding to AgrA
Source: Front Pharmacol. 2024 Apr 10;15:1365815. doi: 10.3389/fphar.2024.1365815 (PMC11039898; doi:10.3389/fphar.2024.1365815)
Supplement: Supplementary file 1 [file DataSheet1.PDF]

Supplementary Material

Physalin H, physalin B, and isophysalin B suppress the quorum-sensing function of *Staphylococcus aureus* by binding to AgrA

Junpei Yamaguchi, Teruhisa Manome, Yasumasa Hara, Yuriko Yamazaki, Yuumi Matusoka, Masami Ishibashi and Akiko Takaya*

***Correspondance:** Akiko Takaya, Ph. D.: akiko@faculty.chiba-u.jp

1 SUPPLEMENTARY TABLES

Table S1. Oligonucleotide sequences used in this study

Primers	Sequence (5'—3')
BamHI-AgrA-F	ACGGATCCGATAATAGCGTTGAAACGATTG
HindIII-AgrA-R	AATTAAGCTTTTATATTTTTTTAACGTTTCTCACC
gyrB-F	CGCAGGCGATTTTACCATTA
gyrB-R	GCTTTCGCTAGATCAAAGTCG
RNAIII-F	GCCATCCCAACTTAATAACCA
RNAIII-R	TGTTGTTTACGATAGCTTACATGC
agrA-F	TGATAATCCTTATGAGGTGCTTGA
agrA-R	CACTGTGACTCGTAACGAAAATAAT
hla-F	CCCGGTATATGGCAATCAAC
hla-R	GGTAGTCATCACGAACTCGT
psma-F	ATCATCGCTGGCATCA
psma-R	AAGACCTCCTTTGTTTGTTATG
Cy5-agrP3-F	AAAACATGCTAAAAGCATTTATTTTCCAATTTTTCTTAACTAGTCGTTTT<br>TTATTCTTAACTGTAAATTTTTTTATGTTAAAATATTAAATACAAATTAC
Cy5-agrP3-R	GTAATTTGTATTTAATATTTTAACATAAAAAAATTTACAGTTAAGAATAAA<br>AAACGACTAGTTAAGAAAAATTGGAAAATAAATGCTTTTAGCATGTTTT
Cy5-LytTR-F	AAATACTTAACTGTTAAAT
Cy5-LytTR-R	ATTTAACAGTTAAGTATTT

Table S2. Physicochemical properties of physalins used in this study

PS	Compound name	MW	Fraction Csp3	H-bond acceptors	Molar Refractivity	TPSA	Mol-LogP
1	physalin H	562.99	0.79	10	131.25	145.66	0.96
2	physalin F	526.53	0.79	10	124.27	137.96	0.76
3	physalin B	510.53	0.71	9	124.78	125.43	1.5
4	isophysalin B	510.53	0.71	9	124.78	125.43	1.5
5	5α-hydroperoxy -6,7-didehydro-5,6-dihydrophysalin B	542.53	0.71	11	127.48	154.89	1.02
6	physalin K	558.53	0.79	12	126.51	164.12	-0.32
7	physalin D	544.55	0.79	11	127.62	165.89	-0.27
8	physalin XII	558.53	0.75	12	127.82	182.96	-1.09
9	physalin G	526.53	0.71	10	125.95	145.66	0.52

2 SUPPLEMENTARY FIGURES

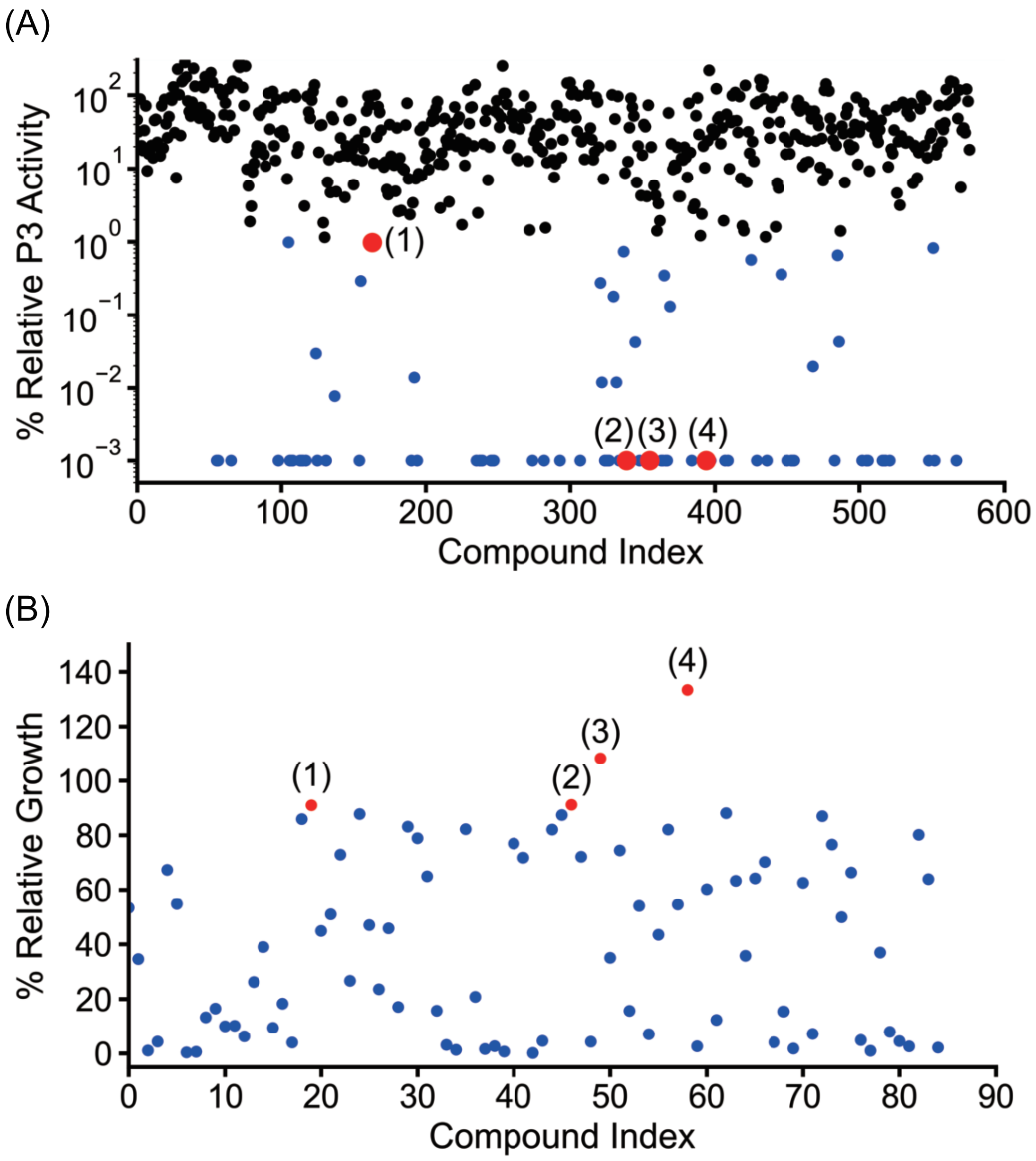

Figure S1. The result of screening of Agr-QS suppression compounds.**(A)** Relative P3 promoter activity in 24 hours of incubation for 577 compounds at screening. Relative P3 promoter activity above 1% is shown in black, less than 1% in blue. **(B)** Relative growth in 24 hours of incubation for 85 compounds at screening. The four hit compounds are shown in red. The four hit compounds (red) are (1) lasidiol *p*-methoxybenzoate, (2) physalin H, (3) ovatodiolide, and (4) ceanothic acid.

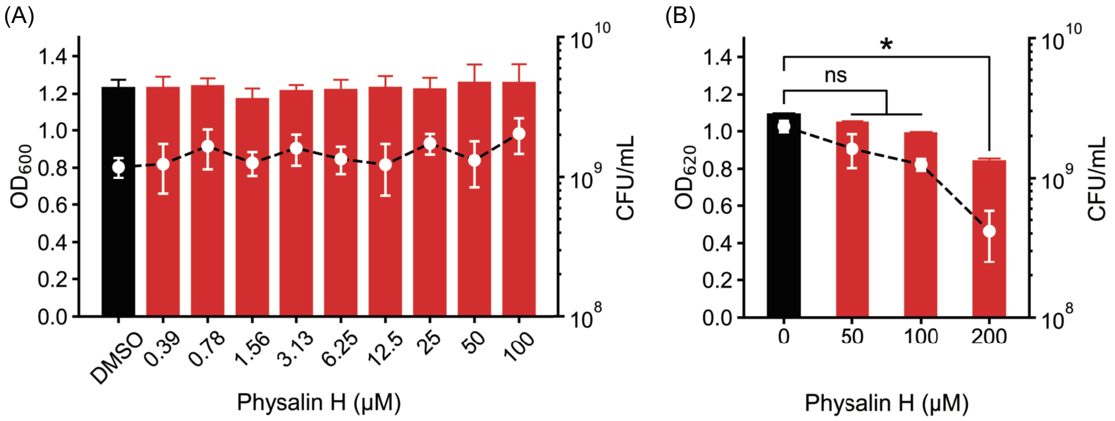

Figure S2. The correlation between OD (bar) and colony-forming unit (CFU) (white circle) in the presence of physalin H at various concentrations. **(A)** OD_{600} and CFU of *agr*::P3-*luc* strain when cultured for 24 h added to media containing serial concentrations of physalin H. **(B)** OD_{620} and CFU after 24 h of culture with LAC strain at 2.5×10^5 CFU/mL and 50, 100, 200 μM physalin H and 1% DMSO (n=3). Data are presented as the mean ± SD. *$p < 0.05$, ns, not significantly different, unpaired t-test.

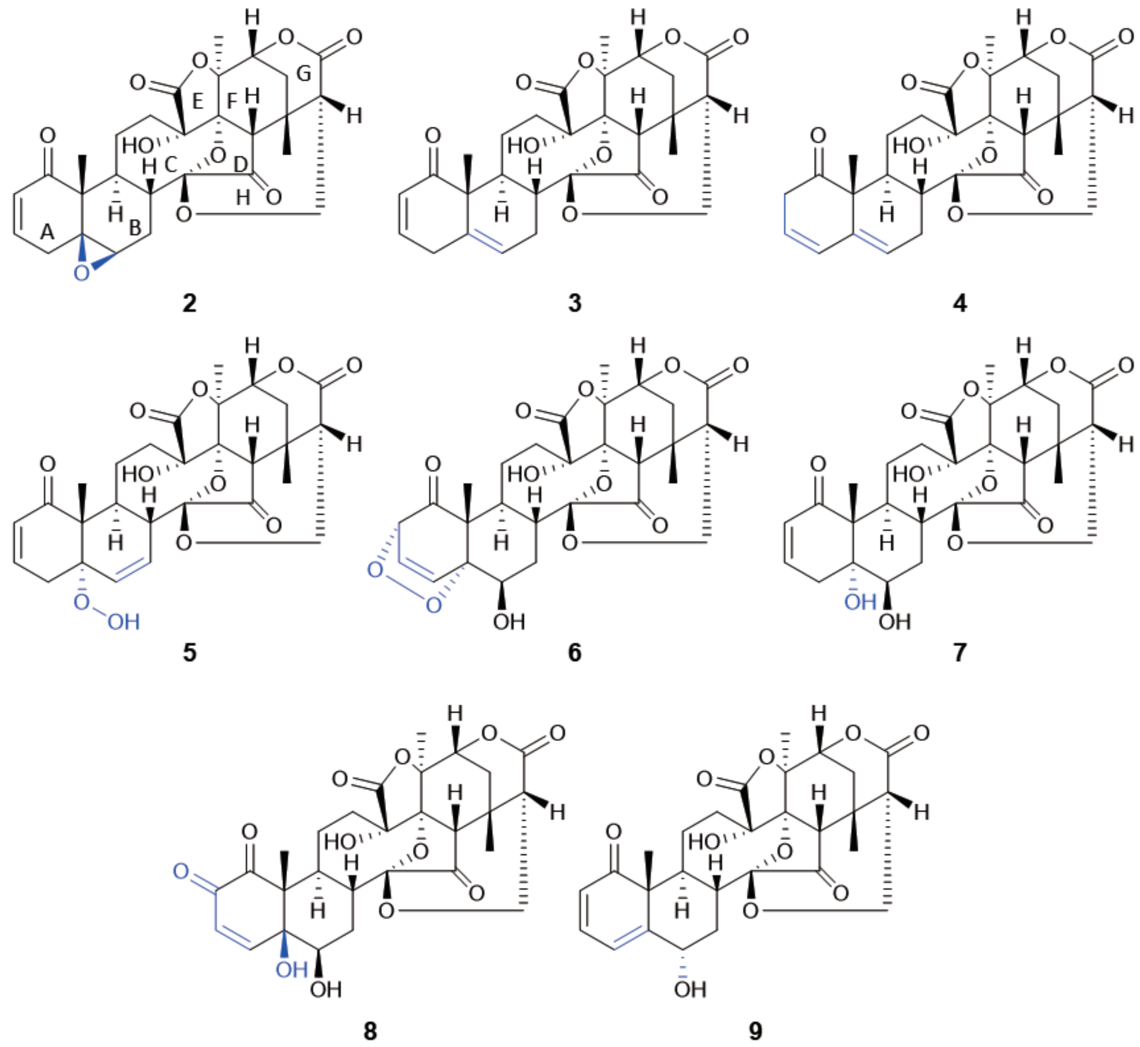

Figure S3. The structures of physalins, PSs2–9, isolated from *Physalis minima*. The blue part in each structure indicates a different part from the structure of physalin H.

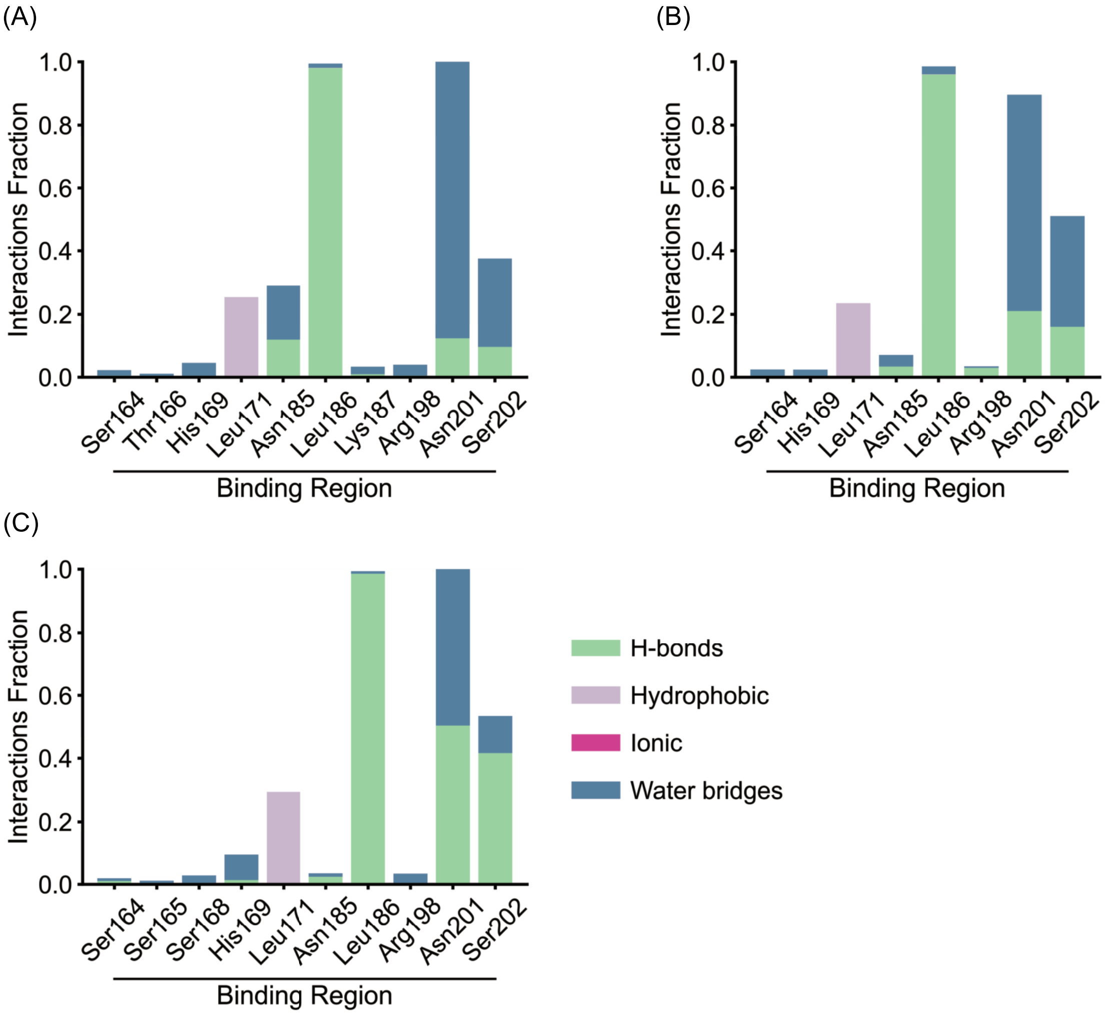

Figure S4. Interaction analysis with AgrA residues in molecular dynamics simulations of PS2 (A), PS3 (B), and PS4 (C).
